# Preclinical study of a self-expanding pulmonary valve for the treatment of pulmonary valve disease

**DOI:** 10.1093/rb/rbaa035

**Published:** 2020-08-22

**Authors:** Dajun Kuang, Yang Lei, Li Yang, Yunbing Wang

**Affiliations:** 1 National Engineering Research Center for Biomaterials, Sichuan University, No 29 Wangjiang Road, Chengdu 610064, China; 2 Venus Medtech (Hangzhou) Inc., 88 Jiangling Road, Hangzhou 311053, China

**Keywords:** pulmonary valve, self-expanding, double bell-shaped, anti-calcification, preclinical

## Abstract

In the past decade, balloon-expandable percutaneous pulmonary valves have been developed and applied in clinical practice. However, all the existing products of pulmonary artery interventional valves in the market have a straight structure design, and they require a preset support frame and balloon expansion. This shape design of the valve limits the application range. In addition, the age of the population with pulmonary artery disease is generally low, and the existing products cannot meet the needs of anti-calcification properties and valve material durability. In this study, through optimization of the support frame and leaflet design, a self-expanding pulmonary valve product with a double bell-shaped frame was designed to improve the match of the valve and the implantation site. A loading and deployment study showed that the biomaterial of the valve was not damaged after being compressed. Pulsatile flow and fatigue *in vitro* tests showed that the fabricated pulmonary valve met the hydrodynamic requirements after 2 × 10^8^ accelerated fatigue cycles. The safety and efficacy of the pulmonary valve product were demonstrated in studies of pulmonary valve implantation in 11 pigs. Angiography and echocardiography showed that the pulmonary valves were implanted in a good position, and they had normal closure and acceptable valvular regurgitation. The 180 days’ implantation results showed that the calcium content was 0.31–1.39 mg/g in the anti-calcification treatment group, which was significantly lower than that in the control valve without anti-calcification treatment (16.69 mg/g). Our new interventional pulmonary valve product was ready for clinical trials and product registration.

## Introduction

At present, patients with pulmonary artery regurgitation and pulmonary stenosis have a high incidence of right-sided heart disease. Among them, tetralogy of Fallot (TOF) is the most common congenital heart disease with right ventricular outflow tract (RVOT) stenosis [1]. The most common age of patients treated with TOF surgery is 6 months to 3 years [2]. In many TOF patients, the RVOT are dilated during surgery to widen a narrow pulmonary artery or valve [2, 3]. However, severe pulmonary regurgitation might occur after surgery. Severe pulmonary regurgitation may lead to a large increase in the right ventricular (RV) volume load, which causes enlargement of the RV cavity in the long term, followed by significantly decreased exercise tolerance and even arrhythmia.

Traditional surgical thoracotomy is likely to cause severe pulmonary regurgitation, which leads to RV enlargement and arrhythmia. Further treatment requires second (or additional) thoracotomy, and although the operation is not only difficult, it has high risk and mortality [4]. With the development of technology, minimally invasive interventional valve replacement surgery has substantially improved and has been widely applied in clinics. Bonhoeffer *et al*. [5] performed the first transcatheter pulmonary valve implantation in 2000. The application of interventional surgery not only effectively terminates massive pulmonary regurgitation, and improves the pulmonary tissue blood circulation and RV function, but it also avoids the need for re-thoracotomy [6]. The pulmonary Melody^R^ valve from Medtronic was first approved by FDA for marketing in 2015, followed by the pulmonary Sapien^TM^ valve from Edwards.

The current artificial biological pulmonary valves, the Sapien^TM^ valve and the Melody^R^ valve, are balloon-expandable and with straight-type support frames [7–9]. When the artificial valve is implanted, a support frame is first placed in conduit to fix the valve [10]. This surgical strategy is expensive. In addition to the valve, a support frame and double-balloon catheters are required. Besides, the shape of the valve is not suitable for congenital heart disease patients with RVOT. The pulmonary valve product developed by us does not require a pre-implantation support frame and balloon catheter, and thus, it significantly reduces the risk for the patients.

In addition, as younger patients are being diagnosed with pulmonary artery valve disease, bioprosthetic valves with enhanced anti-calcification property and longer lifespan are urgently needed [6, 11, 12]. Therefore, the development of interventional pulmonary valve implantation system with high resistance to calcification and excellent durability has become an inevitable trend in research on the treatment of pulmonary valve diseases.

The purpose of this study was to develop a new interventional self-expanding pulmonary valve system for the treatment of pulmonary regurgitation and stenosis. Our pulmonary valve product was designed to fit the anatomy, specifically of dilated outflow tracts, to restore the pulmonary valve conduit function. It comprised a self-expanding shape memory alloy multi-level support frame with a tri-leaflet porcine pericardium tissue valve, a 19-, 22- or 24-Fr catheter delivery system, a single use/disposable loading system with crimping devices and an introducer. By optimization of the design of the support frame, leaflet and anti-calcification treatment, we aimed to expand the range of use of the product in the population with pulmonary valve disease and to prolong its service life.

## Materials and methods

### Design and preparation of the shape memory alloy support frame

The support frame was made of laser-cut nickel-titanium (Ni-Ti) thin tubes. After polishing and heat treatment, the required diameter size was obtained. The mesh design at the INFLOW (blood flow inlet) was smaller, and the top of the flow inlet was concave in shape to avoid pressing the chamber and causing other cardiac complications. Through SolidWorks modeling and finite element analysis, the radial force at the entrance could support the original heart valve so that it would not fall off and other functions of the heart were not affected due to excessive stress. The mesh design at the OUTFLOW (blood flow outlet) was larger so that the radial force at the outlet of the support frame provided support on the blood vessel. The overall shape of the support frame was verified by SolidWorks simulation analysis.

### Mechanical properties of valve support frame

Before the test, the external dimension inspection and visual inspection were carried out for the frame. The frame was compressed at the rate of 1 mm/s. In this process, the radial resistive force (RRF) value at the middle straight section compression amount of 4 mm and the chronic outward force (COF) value of the middle straight section compression amount of 2 mm were recorded.

### Preparation of biological valves

Fresh porcine pericardia were collected and washed. Glutaraldehyde crosslinking and anti-calcification treatment were performed. In view of several major factors that cause calcification, including glutaraldehyde aldehyde group residue, elastin degradation, immunogenicity and phospholipid residue, our product adopted a variety of comprehensive methods to optimize the anti-calcification treatment technology. The pericardia were cut with a laser to design the shape of the valve leaflet. The porcine pericardia leaflets were sutured to the Ni-Ti alloy frames.

### Mechanical properties of porcine pericardium

Pericardia measuring 10 × 50 mm were cut with laser and numbered, and the thickness of each pericardium sample was measured and recorded. Pericardia were sewed together in pairs at the end of 10 mm to form the suture sample without support. Pericardia were also sutured together with a 0.4 mm stainless steel wire to form the sutured sample with support.

### Loading and deployment study

By simulating the conditional state of the product in actual use, the performance of the product in loading and deployment could be quantitatively tested to meet the use requirements. Load performance was tested at 0°C (ice water). An appropriate amount of water and ice was placed in an ice bucket to form ice–water mixture. The whole product was placed into the ice bucket for 10 min. The valve support frame was continually compressed through the high-pressure injection pump. The circumference of the valve support frame was measured with a caliper, and the output pressure of the high-pressure injection pump was recorded. Three samples with the same specification were kept in the compressed state for 10 min and 1 h, and then the bioprosthetic heart valve was removed and a microscope was used to analyse whether the crease had caused damage to the tissue fiber.

### Pulsatile flow and fatigue in vitro tests

The treated pericardia were tailored on the scaffold to form pulmonary valves. The hydrodynamic performance test was performed on an *in vitro* pulsatile flow instrument (HDT-500 Pulse Duplicator, BDC Laboratories, CO, USA) and a fatigue instrument (DuraPulse™ Heart Valve Durability Test Instrument, TA Instruments, USA) under the specific conditions according to ISO 5840-3:2013, Cardiovascular Implants-Cardiac Valve Prostheses-Part 3: Heart valve substitutes implanted by transcatheter techniques. The effective orifice area and the valve regurgitation ratio were measured by the Statys^®^ HDT Software (HDT-500 Pulse Duplicator, BDC Laboratories, CO, USA).

### Evaluation of pulmonary artery implantation in preclinical studies

Animal study received the proper ethical oversight. The study complied with all institutional and national requirements for the care and use of laboratory animals and received animal care and use committee approval from Shanghai Zhongshan Hospital. Pigs were used in our preclinical study since they have similar cardiovascular system structures as humans. This animal study was performed to observe the acute and chronic health statuses of pigs after valve implantation and to assess long-term safety and operability of the percutaneous pulmonary valve. A total of 11 adult white pigs were used, and the brief study design is shown in Table 1. The animals used in this study were 5 ± 2 months old and weighed 45 ± 5 kg. The experimental animals were divided into the following five groups: acute, 30 days’ post-implantation, 90 days’ post-implantation, 180 days’ post-implantation and 360 days’ post-implantation.

**Table 1.** Detailed information of 11 adult white pigs used in the study

No.	Group	Time until death	Animal No.	Valve type
1	Acute	Instant	P555	Anti-calcification valve
2	30 (±3)	30 (±3)	P489	Anti-calcification valve
3	90 (±7)	90 (±7)	P556	Anti-calcification valve
4	90 (±7)	90 (±7)	P570	Anti-calcification valve
5	90 (±7)	90 (±7)	P490	Anti-calcification valve
6	180 (±10)	180 (±10)	1946	Control valve
7	180 (±10)	180 (±10)	1948	Anti-calcification valve
8	180 (±10)	180 (±10)	1949	Anti-calcification valve
9	180 (±10)	180 (±10)	1952	Anti-calcification valve
10	180 (±10)	180 (±10)	1953	Anti-calcification valve
11	360 (±10)	360 (±10)	P915	Control valve

The operation process is described below. One day before the procedure, aspirin (100 mg) and cefradine were given. General anesthesia and aseptic techniques were adopted during the operation. A mixture of isoflurane and oxygen was continuously provided to maintain anesthesia. This minimally invasive approach was performed under general anesthesia along with utilizing angiography for visualization. The left femoral artery was punctured to embed the 6 Fr sheath and the right femoral vein was punctured to embed the 20 Fr sheath. The 7-Fr arrow balloon catheter was inserted into the right ventricle and the main pulmonary artery (MPA) to assess RV systolic function and pulmonary valvular insufficiency. The inner diameter of the RVOT-MPA was measured to determine the position. Before the procedure, a multi-track catheter was inserted through a sheath embedded in the right femoral vein to the RVOT-MPA to test hemodynamics, pressure of the right atrium, RV, MPA and a branch of the pulmonary artery, as well as the transvalvular pressure gradient. The Amplatz super stiff guide wire route was described as follows: right femoral vein-inferior vena cava-right atrium-RV-pulmonary artery-distal part of the left pulmonary artery. The balloon catheter was inserted to the RVOT-MPA through the guidewire and the 5-Fr pigtail catheter was advanced to the ascending aortic sinus through a 6-Fr introducer. Angiographic measurements of the intended implantation site were obtained to assess if the conduit was suitable for implantation. The valve was deployed slowly, and the position was monitored under fluoroscopy. During implantation, continuous angiographic measurements were performed to obtain the clinical and hemodynamics results. If the valve was not deployed well enough, dilation with a high-pressure balloon was performed. A pulmonary artery angiogram was performed to assess regurgitation. All segmentation images were recorded by cardiac ultrasound. The RVOT, annular diameter, MPA inner diameter and length and hemodynamics were measured repeatedly before the procedure. During the procedure, all steps were monitored under fluoroscopy and operation results were recorded immediately.

Post-procedure management and follow-up are described below. After implantation of the valve, pulmonary angiography and ultrasound cardiography were used to monitor hemodynamics, regurgitation, transvalvular pressure gradient and implantation position. Acute stage experimental pigs were euthanized. After withdrawing the apparatus in the long-term experimental pigs, the incision was sutured and the puncture was pressed until bleeding stopped. The pigs were sent to an experimental animal room after surgery and their post-recovery were observed. Aspirin (100 mg) was used daily until the experimental endpoint was reached in each pig. Hemodynamics, regurgitation, transvalvular pressure gradient and implantation position were observed. After angiographic and ultrasound cardiography examination, euthanasia was performed in the experimental pigs. The valves were removed to carry out general and electron microscopy examinations. The heart, lung, liver, kidney and spleen were removed for general and pathological examinations.

### Calcification study in the animal test

The weight of six experimental pigs (animal numbers 1946, 1948, 1949, 1952, 1953 and P915) for the calcification test was about 45 kg, and six pulmonary valves were implanted in these pigs. The pigs were dissected after the growth cycles of 6 months and 12 months. The valve was removed and the amount of calcium was determined.

## Results

### Design and preparation of Ni-Ti memory alloy support frame

**Figure 1.**
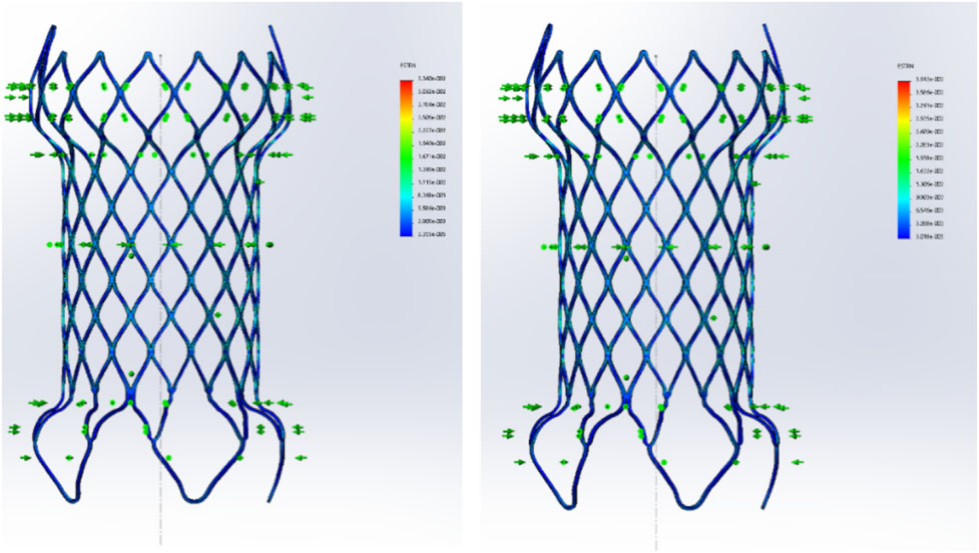
Deformation of the first load and the second load in finite element analysis of the valve support frame.

The Ni-Ti valve support frame needs to be compressed into a smaller diameter with cylindrical shape in ice water to be smoothly inserted into a simulated blood vessel. The inner diameter of the simulated blood vessel was selected based on the size after compression of the middle section of the support frame diameter by about 10%. The finite element analysis used a cylindrical coordinate system, as shown in Fig. 1. The boundary conditions and loads were set as follows: the first load step exerted a radial compression displacement on the lotus-shaped nodes in the inflow section of the support frame, the middle diamond-shaped grid in the middle section and the middle section of the last row of diamond-shaped grid in the outflow section from the bottom to the top. The circumferential and axial directions were not restricted. The maximum strain of first load was 3.340%. In the second load step, pulsating loading with amplitude 2% was performed. In order to facilitate the analysis and prevent the axial and circumferential movement of the support during the analysis, circumferential limit treatment was carried out for 15 nodes in the middle section.

**Figure 2.**
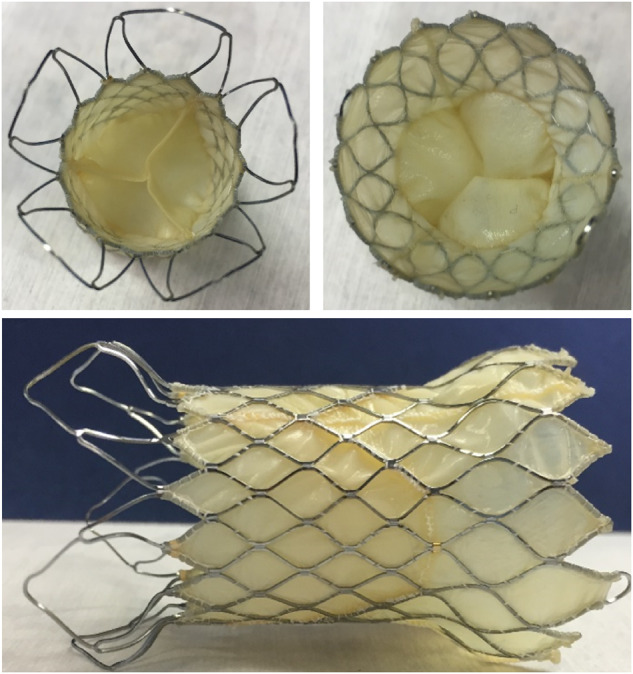
Photos of the self-expanding pulmonary valve product.

According to the results of the finite element analysis, the maximum strain produced by the largest support frame was 3.912%, the average strain was 3.626% and the strain amplitude was 0.286%. The maximum strain node was located at the diamond-shaped grid root at the end where the inflow end of the support frame was excessive toward the middle straight section of the support frame. The main factors affecting fatigue life were the average stress and strain amplitude of the alternating load. Our optimized design is shown in Fig. 2. The existing pulmonary valve frame was a saddle-shaped cylinder structure. The tip of one end was sharp and dense. During the release of the frame, the sharp top tended to puncture the vessel and increase the difficulty and risk of the operation. Moreover, the dense structure had a strong blocking effect on blood flow, which affected the smoothness of blood flow.

### Mechanical properties of valve support frame

Results showed that the RRF and COF of the pulmonary valve stent were within the range specified in the design specifications for pulmonary valve products (25 mmHg ≤ COF ≤ 500 mmHg; RRF ≥ 100 mmHg), indicating that the design and manufacturing process of our pulmonary valve was reasonable and stable.

### Leaflet design and mechanical properties of the pericardium

**Figure 3.**
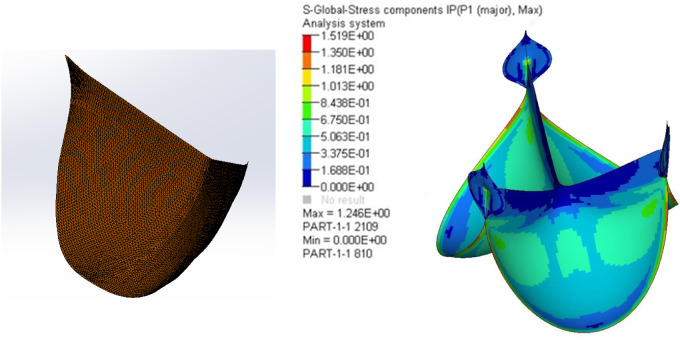
Grid division and stress modulation results obtained from finite element analysis.

The grid division and stress modulation results after finite element analysis are shown in Fig. 3. There was no special stress concentration point and no excessive stress or stress concentration point at the detected suture edge. The maximum stress at the stress point according to finite element analysis was 1.246 MPa. Using verification records, it was noted that the minimum tensile strength of the suture sample without the support frame was 4.27 N/mm^2^, while the minimum tensile strength of the suture sample with the support frame was 4.25 N/mm^2^ (Table 2); both values were greater than the finite element analysis maximum stress, which was about 3.4 times of the maximum stress.

**Table 2.** Summary of the suture strength data

Sample:Tensile strength	Pericardial sample	Suture sample without support frame	Suture sample with support frame
Average（N/mm^2^）	18.011	5.5911	5.9838
Standard deviation	0.84778	1.2472	1.3933
Remarks: FEA (max) = 1.246 Mpa			

### Loading and deployment study

**Figure 4.**
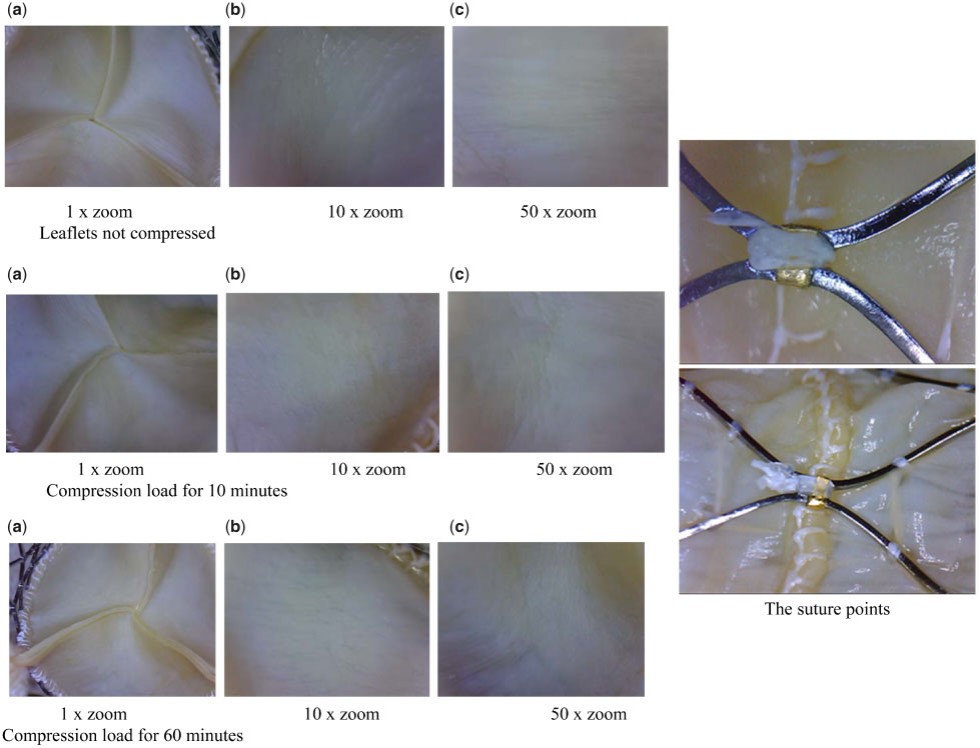
Photos of leaflets with different compression times as well as the suture points.

The biomaterial of the valve and the suture points were not damaged after being compressed, as shown in Fig. 4. Since it was loaded on site during the actual operation, the compression time was <1 h, and it was usually implanted in the body within 20 min. The biomaterial of the valve and the suture technology met the requirements for use of the product.

### Pulsatile flow and fatigue in vitro tests

According to the standard regulations, pulsating flow properties were detected and analysed under 12 test conditions for pulmonary artery valves with diameters compressed by 2 mm and 4 mm. Under various physiological conditions, the valve could close and open completely. Under the test conditions of heart rate of 70 beats/min, mean arterial pressure of 20 mmHg and mean cardiac output of 5 L/min, the results were in accordance with ISO 5840-3:2013, Cardiovascular Implants-Cardiac Valve Prostheses-Part 3: Heart valve substitutes implanted by transcatheter techniques. According to the criteria of the standard of artificial heart valve, the variation trend of the results under different test conditions conformed to the general rule of Bernoulli equation; this showed that the design and manufacturing process of the newly produced pulmonary valve were reasonable and stable.

**Figure 5.**
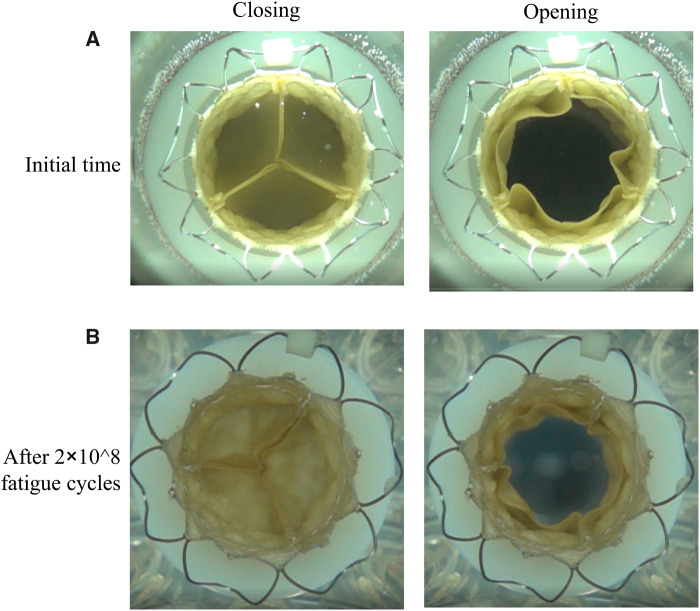
Comparison of leaflets after the *in vitro* accelerated fatigue test. The closing and opening statuses of leaflets during the *in vitro* pulsatile flow test before the *in vitro* accelerated fatigue test (**A**) and after *in vitro* 2 × 10^8^ accelerated fatigue cycles (**B**).

After 2 × 10^8^ accelerated fatigue cycles, the leaflets of the pulmonary valve showed no cracks and breaks, and they could be completely closed without the backflow (Fig. 5). The leaflets could also be completely opened after the *in vitro* accelerated fatigue test. These results indicated that the fabricated pulmonary valve met the hydrodynamic requirements after 2 × 10^8^ accelerated fatigue cycles.

### Evaluation of pulmonary artery implantation in preclinical pigs

**Figure 6.**
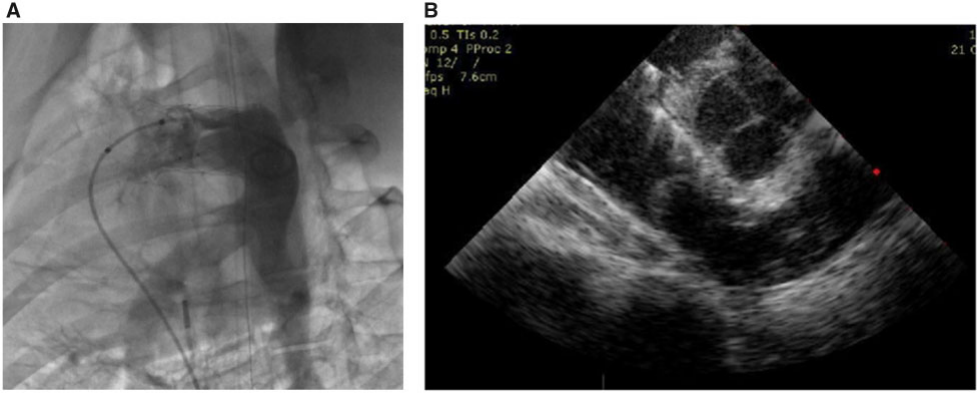
Angiography (**A**) and echocardiography (**B**) showed that the valve was positioned well and there was no obvious regurgitation.

All valves were successful implanted into 11 experimental pigs. The average duration of the operation was 120 ± 30 min. Angiography and echocardiography were performed promptly after the operation, and they showed that the valves were positioned well and there was no obvious regurgitation (Fig. 6). The inspection at each observation endpoint after the operation also showed that all valves were positioned well and there was no obvious regurgitation.

**Figure 7.**
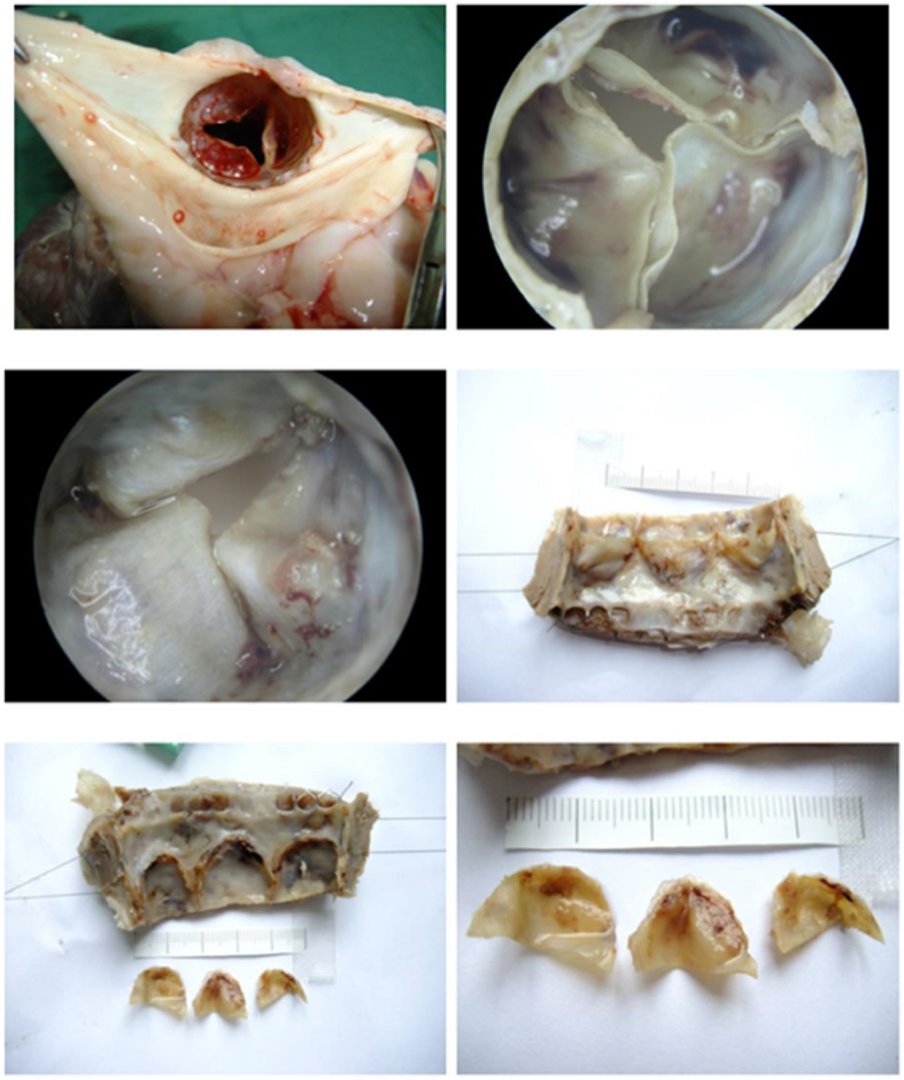
Representative photos of the anti-calcification valves after 180 days’ implantation.

For general observations and histopathology, calcification did not appear in experimental pigs implanted with anti-calcification valves for 180 days (Fig. 7). The implanted valves showed relatively good positioning and large tension in the animals upon post-mortem inspection. The valve support frames were complete and free of breakage. The surface of the valve was smooth and free of thrombus traces.

**Figure 8.**
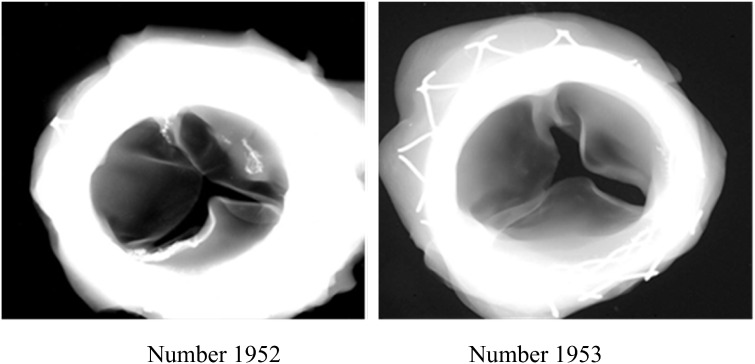
Representative photos of main organs from the pigs implanted with anti-calcification valves after 180 days.

Main organs, such as liver, heart, spleen, lung and kidney, and their respective sections from the pigs implanted with anti-calcification valves after 180 days were generally normal and free of ischemic necrosis tissues caused by embolism (Fig. 8).

To sum up, all pigs implanted with anti-calcification valves were free of thrombus and showed complete valve structures, smooth valve surfaces and low calcification. Transcatheter implanted valve products were successful in 11 laboratory animals. The short-term follow-up after the operation showed the safety and effectiveness of the implanted device.

### Calcium content of the valve in the animal test

**Figure 9.**
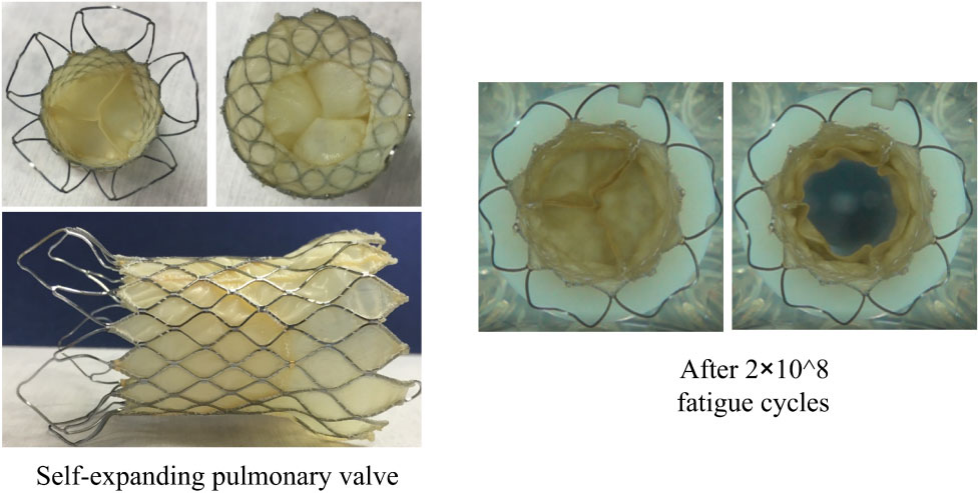
X-rays were performed and they showed no obvious calcification.

X-rays were performed for representative explanted valves, and they showed no obvious calcification (Fig. 9). Sonography and anatomy showed that the anti-calcification valves (Number 1952 and Number 1953) remained intact, with normal activity and good performance.

The detailed calcium content in each implanted valve is shown in Table 3. The calcium contents of the control valve at 6 and 12 months of growth reached 16.69 and 63.7 mg/g, which were much higher than the calcium content of the 6 months’ anti-calcification valves at 0.31–1.39 mg/g (Table 3).

**Table 3.** Valve leaflet calcium test results

Animal number	Valve type	Implantation time (days)	Calcium content (mg/g)		Test animal
			Atomic absorption	Emission spectrometry	
1946	Control valve without anti-calcification treatment	180 (±10)	16.69	24.51	Pig
1948	Anti-calcification valve	180 (±10)	1.39	2.77	Pig
1949	Anti-calcification valve	180 (±10)	1.92	4.95	Pig
1952	Anti-calcification valve	180 (±10)	0.65	4.55	Pig
1953	Anti-calcification valve	180 (±10)	0.31	2.14	Pig
P915	Valve without anti-calcification treatment	360 (±10)	63.7		Pig

## Discussion

The implanted anatomical position of the pulmonary valve is complicated, as it requires the valve not only to open the stenosis and calcification but also to not compress the coronary artery vessels [13]. The risk of pulmonary artery rupture and the RVOT rupture should be considered. Therefore, to design the shape of the valve frame, the anatomical structure data of the implantation position of the human pulmonary artery valve should be used to simulate and reconstruct a three-dimensional model on the computer, and the three-dimensional design software should be used to perform simulation implantation analysis to determine whether the above risks can be avoided.

In this study, we first used a new design for pulmonary artery support frame. The interventional artificial pulmonary valve product developed in this study was a new type of self-expanding heart valve. The valve was installed in a self-expanding Ni-Ti memory alloy support frame. The middle part of the support frame was the straight tube type, and the upper and lower parts were horn-type expansion. This type of support frame was designed to prevent valve movement after implantation [14, 15]. Compared with existing pulmonary valve products, in the current study the design of the frame outflow segment avoided puncture of the MPA or pulmonary artery branch vessels during the implantation process of the stent, which reduced the difficulty of surgery and avoided the risk of surgery. Besides, the double bell-shaped frame was not easy to shift after implantation.

Other pulmonary valves, Sapien^TM^ valve and Melody^R^ valve in the market, were straight valves for pre-stented artery and could only treat around 20% patients [16, 17]. The pulmonary valve product developed by us fitted the anatomy to the maximum extent and had a unibody design. The outflow had a smooth rounded frame for ultimate artery protection. The middle section had a frame for housing the valves. Special length could be designed and made quickly. The inflow was conformed to the RVOT and it tapered at the end for artery protection. The flared outflow end secured anchoring at pulmonary artery bifurcation. Radiopaque markers indicated the positions of anchoring at the top and the valve location. The uncovered end maintained pulmonary artery patency. The flared and covered inflow end conformed to the dilated RVOT. The valve sizes were chosen on the basis of patient’s CT, MRI and sizing balloon measurements. 3D printing was performed to generate a model for the patient anatomy. The valve was deployed into the model for best fit verification.

The second major component of the pulmonary valve product was the leaflet made of pericardium. The mechanical properties of porcine pericardium were simulated and tested. Our results showed that the fracture tensile force and tensile strength of the suture sample without support frames were basically the same as those of the suture sample with support frames. Through the suture technique, pericardia were bound on the support frame so that the tension was dispersed on the support frame, thus reducing the tension borne on the pinhole. Calcification of the leaflet limits the durability of the valve product [18, 19]. Calcification of valve material is an important factor affecting the service life of the biological valve. Calcification greatly affects the normal work of the valve, which is a major issue that needs to be solved urgently. Long-term research has been carried out on the anti-calcification of the valve and some progress has been made, but the calcification problem still exists. The main reason for this occurrence may be that the understanding of the calcification mechanism is not complete. We believe that the mechanisms of biological valve calcification mainly include the following: (i) glutaraldehyde aldehyde group residue [20], (ii) elastin degradation [21–23], (iii) tissue phospholipid residue [24], (iv) glycosaminoglycan degradation [25, 26], (v) immunogenicity of heterogeneous tissues [27], (vi) residual cells [28] and (vii) difficulty in endothelialization [29–31]. Our anti-calcification strategy mainly targets the key factors of glutaraldehyde aldehyde group residue, elastin degradation and tissue phospholipid residue. Compared with the control valve without anti-calcification treatment, the calcium contents of our anti-calcification valves were decreased by 12 times. The angled leaflet design reduced stress concentration. For the treatment of pericardial materials, the anti-calcification effect was greatly improved.

To sum up, this study mainly summarized the basic data of our research and the development of self-expanding interventional pulmonary valve products. The available data showed that the properties of the newly developed pulmonary valve product had met the requirements of product development. This provided the basis for clinical trials and product registration.

## Conclusions

The performance of the newly developed interventional pulmonary valve product met the technical requirements. Our new interventional pulmonary valve product was ready for clinical trials and product registration.

## Funding

This work was supported by the National Key Research and Development Programs (SQ2019YFC110002).

*Conflict of interest statement*. None declared.
